# Overview of the Sustainable Valorization of Using Waste and By-Products in Grain Processing

**DOI:** 10.3390/foods12203770

**Published:** 2023-10-13

**Authors:** Cristina-Anca Danciu, Anca Tulbure, Mirela-Aurora Stanciu, Iuliana Antonie, Ciprian Capatana, Mihai Victor Zerbeș, Ramona Giurea, Elena Cristina Rada

**Affiliations:** 1 Food Industry and Environmental Protection, Lucian Blaga University of Sibiu, 7-9 Dr. Ion Ratiu Street, 550012 Sibiu, Romania; cristina.danciu@ulbsibiu.ro (C.-A.D.); mirela.stanciu@ulbsibiu.ro (M.-A.S.); iuliana.antonie@ulbsibiu.ro (I.A.); ciprian.capatana@ulbsibiu.ro (C.C.); 2 Department of Industrial Engineering and Management, Lucian Blaga University of Sibiu, 4 Emil Cioran Street, 550025 Sibiu, Romania; mihai.zerbes@ulbsibiu.ro (M.V.Z.); ramona.giurea@ulbsibiu.ro (R.G.); 3 Department of Theoretical and Applied Sciences, University of Insubria, 46 Via G.B. Vico, 21100 Varese, Italy; elena.rada@uninsubria.it

**Keywords:** grain waste, grain by-products, food industry, product quality assurance, sustainable valorization, circular economy

## Abstract

In an increasingly resource-constrained era, using waste and by-products from grain processing has a wide appeal. This is due to the nutritive value and economic aspects of this process and due to its compatibility with the trend towards more sustainable food systems. Following the fundamentals of circular economy, a current need is the effective utilization of grain waste and by-products for conversion into value-added products in the food industry. The aim of this study is twofold: (1) using bibliometrics and the literature found in various databases, we aim to understand the progress of valorizing grain waste and by-products in human nutrition. The literature within various databases, namely, Google Scholar, Web of Science, and Elsevier Scopus, has been evaluated for its merits and values. (2) We aim to explore knowledge-based strategies by reviewing the literature concerning the possible use of grain waste and by-products for the food processing industry, reducing the burden on virgin raw materials. The review allowed us to unlock the latest advances in upcycling side streams and waste from the grain processing industry.

## 1. Introduction

In the era of anthropogenic waste generation, an exponential increase in the need to fulfill the nutritional basics of humans has prompted the scientific community to study emerging themes and hot issues regarding this global challenge [1,2,3,4,5,6].

Worldwide, the human diet is supported by staple cereals obtained from seeds of the Gramineae family, such as wheat (*Triticum* spp.), corn or maize (*Zea* spp.), barley (*Hordeum* spp.), rice (*Oryza* spp.), rye (*Secale* spp.), oat (*Avena* spp.), millet (*Pennisetum* spp.), sorghum (*Sorghum* spp.), and a hybrid of wheat and rye, namely, triticale (Triti-cosecale Wittmack). Cereal grains contribute significantly to the global food pool in terms of global food security and nutrition. Most cereals are a staple source for various amounts of proteins, fats, minerals, and vitamins and are an important provider of dietary energy [7]. In total, the percentage of dietary energy provided by cereals appears to have remained relatively the same over time, representing about 50% of dietary energy supply [8,9]. Worldwide, for over one billion people, maize is a staple, and its grain energy contribution to the diet can exceed 50% [7]. In second place, in terms of cultivated area and human consumption, wheat represents almost 20% of the total dietary calories and proteins globally [10]. Rice contributes 20% of global calories and is an important source of minerals and vitamins. Bran contains bioactive phytochemicals and essential food components [11].

Worldwide, corn is the most cultivated and used plant, constituting a basic ingredient in many gastronomic cultures in addition to being important in animal feed, the production of biofuel, and many other industrial uses. As a staple food, it is estimated that its production is over 1136.3 million metric tons (from September 2020 to August 2021), which is much more than wheat production (776.8 million metric tons) or rice production (504.4 million metric tons) [12]. In the latest revised global forecast, according to the FAO, the total production of cereals in 2022 was 2774 million tons, 1.3% less than the previous year [13].

**Figure 1:**
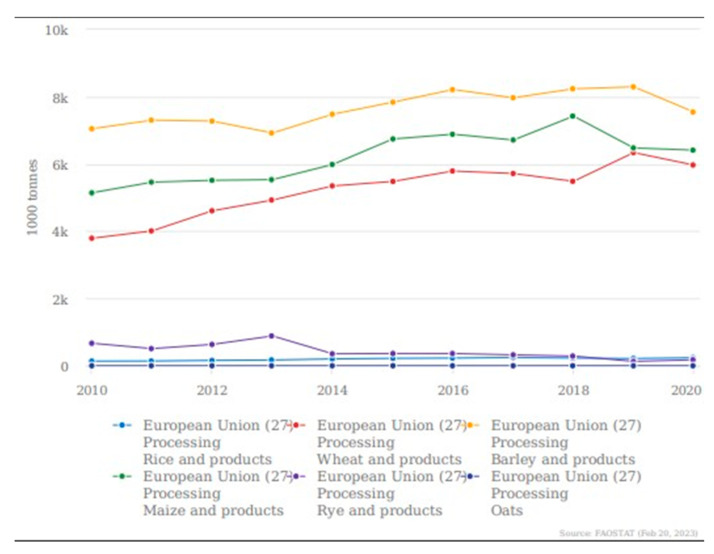
Processing of wheat, maize, rice, rye, barley, oats, and related products in the European Union (27 member states). Source: compiled by authors, based on FAOSTAT online database, 20 February 2023.

The forecast for global cereal utilization in 2022/23 is 2780 million tons. This indicates a decline of 0.6% from the 2021/22 level but is greater than the total cereal production of 2022 [13]. One of the reasons that triggered the bibliographic research is the fact that, according to the latest data published by the FAO in March 2023, this excess of cereal consumption—more than the quantity produced, the demand, and the reserve—was predicted. Therefore, under the conditions of an increased demand for resources to feed a continuously growing population, solutions must be sought for the use of all natural sources, making the most of their potential. With this desire, the United Nations established, in 2015, the Sustainable Development Goal (SDG) 12, to “Ensure sustainable consumption and production patterns”. Target 12.3, which refers to food waste, stipulates that by 2030, per capita global food waste at the retail and consumer levels will be halved, and food losses from the production flow will be reduced along with those from the supply chains, including post-harvest losses. The European Commission considers food waste as a priority area in order to achieve the Sustainable Development Goal target in agreement with the European Circular Economy Action Plan [14]. In addition, the European Commission amended the Waste Framework Directive 2008/98/EC, establishing as mandatory the monitoring and reporting of food waste by member states to create a baseline for monitoring the achievement of food waste reduction objectives and help identify relevant food waste streams to be utilized in a circular economy perspective [14,15,16,17]. In the European Union, the main grains processed from 2010 to 2020 were wheat, maize, rice, rye, barley, oats, and their related products. Figure 1 shows the quantity of each.

Grain processing must and can be a sustainable option to convert waste and by-products into value-added resources for the food industry and for their valorization under the circular economy (CE) concept. Innovative methods, on an industrial scale, for the recovery of food waste—instead of its disposal—must be developed in agreement with CE concepts. Currently, only conventional methods are applied on an industrial scale, providing animal feed, biofuel production, or aerobic/anaerobic treatments, which represents only a partial utilization of cereal processing waste [18,19,20,21,22].

The aim of this study is twofold:

1. Understanding the progress of grain waste and by-products in the valorization of human nutrition, using bibliometrics. The literature in various databases, namely, Google Scholar, Web of Science, and Elsevier Scopus, has been evaluated for its merits and values;
2. Exploration of knowledge-based strategies by reviewing the literature concerning the possible use of grain waste and by-products for the food processing industry, reducing the burden on virgin raw materials. The review allowed us to unlock the latest advances in upcycling side streams and waste from the grain processing industry. An overview of the food industry’s sustainable applications in the recovery and reutilization of waste cereal processing and by-products was also considered.

## 2. Bibliometric Analysis 

**Figure 2:**
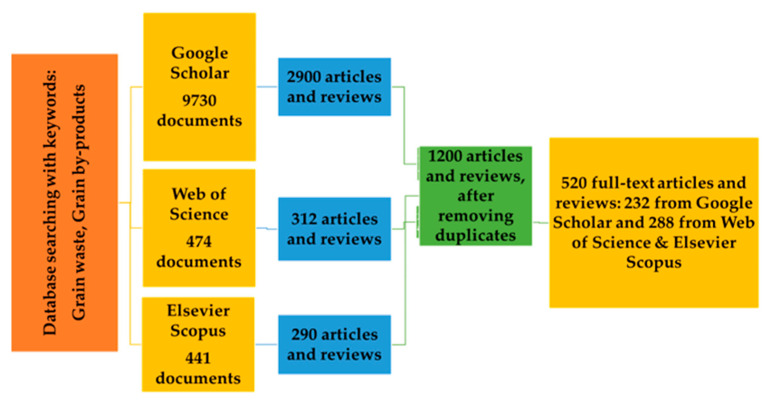
Flowchart representing the selection process of articles and reviews from Google Scholar, Web of Science, and Elsevier Scopus databases.

In this paper, a bibliometric analysis was performed to identify, screen, and analyze published research articles and reviews [23]. The aim was to retrieve and select those pa-pers that investigate and define the current state of the art on grain by-products and grain waste recovery and the ways in which their processing can benefit the food industry. The research trends were evaluated based on papers from Google Scholar^®^, Web of Science^®^ and Scopus^®^ databases, over the last decade (2012 until 2023). To ensure the quality of the research, only peer-reviewed articles were selected. Criteria used for article selection were title, abstract, and keywords. Document type was limited to “articles” and “reviews”. For the research, the terms “Grain AND Waste AND By-products” were used. The list obtained after the initial search was screened by reading the title and abstract (duplicates and articles that only consisted of an abstract were excluded), followed by a full-text reading. All articles and reviews about grain waste and/or grain by-products were included, while other, non-relevant papers, were discarded. After exploring the databases, a flowchart was produced, summarizing the obtained results. This flowchart is shown in Figure 2.

Following the bibliometric analysis, 520 manuscripts of full research articles and reviews were selected; of these, 228 (44%) were published in 2019 or later.

**Figure 3:**
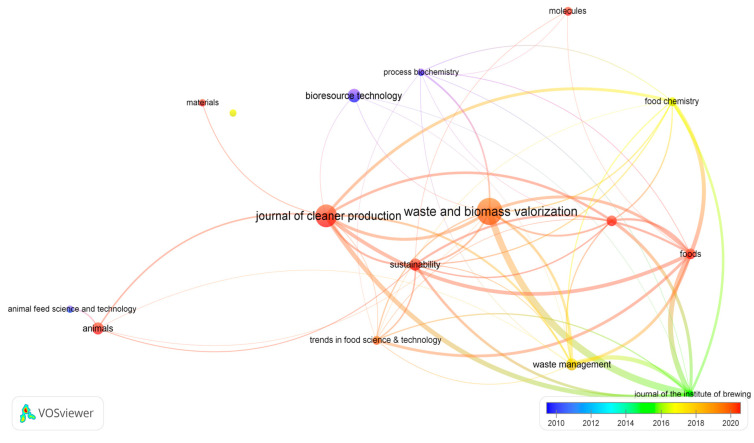
Average publications per year (overlay visualization) related to grain by-products and waste, for the last decade—compiled by authors.

Measurements of the quality and quantity of the scientific production [24] were carried out using VOS Viewer’s science mapping software tool version 1.6.19 [25]. Previously, selected data for VOS Viewer supported file types (Web of Science^®^ and Scopus^®^) that were exported to Microsoft 365 Excel and then saved on .txt format. After a bibliographic coupling analysis (the relatedness of items is based on the number of references they share), the manuscripts were assessed and classified according to the average number of documents per year (for the last decade) and distribution by journals. The results are reported in Figure 3.

There was an increase in the number of articles related to the use of waste resulting from grain processing starting in 2018, indicated by the red circles. The diameter of these circles is proportional to the number of articles published per year in the most productive journals in the field. This is due to the continuous concerns at the level of the European Union and the world regarding the regulations and scientific approach to facilitate the use and valorization of grain waste and by-products from the food chain, without compromising food and feed safety, in compliance with CE concepts.

The top 16 most-productive journals are ranked in Table 1 with the aim of displaying the results more conveniently, not only in terms of the number of articles related to grain waste conversion but also the number of related citations. 

**Figure 4:**
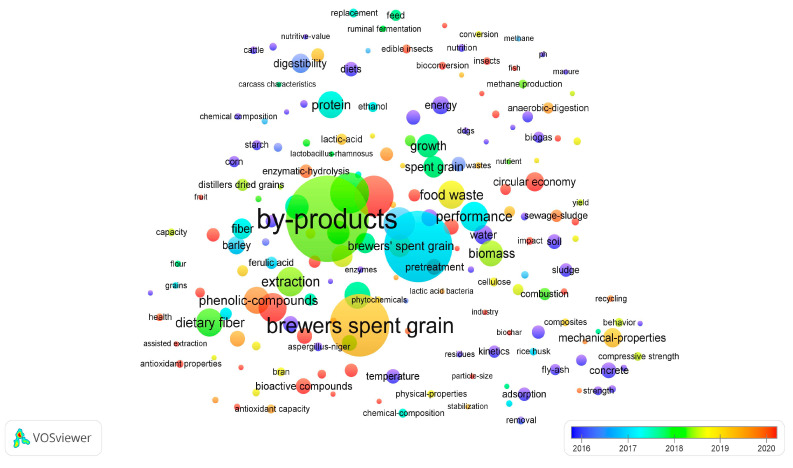
Word cloud based on the main keywords in cereal waste and cereal by-products research documents (overlay visualization); compiled by authors.

A word cloud constructed according to searched keywords is shown in Figure 4. The font size of the given keywords is proportional to the number of times that the keyword appeared in the literature. The most numerous occurrences are naturally represented by the keywords (grain waste and by-products) and the terms used most often in the management of waste resulting from grain processing, such as circular economy, food waste, biomass, brewer’s spent grain, phenolic compounds, dietary fiber, pretreatment, extraction, etc. 

Grain waste and by-products produced across the technological chain can have a negative impact ecologically, socially, and economically if they are not managed using a CE approach and in concordance with the SDGs. Upcycling grain process side streams and waste into ingredients with added value for food confronts the challenge of ensuring increased stability of the food supply chain under conditions of global political insecurity, severe climate change, and continuous demographic growth. As a consequence, the academic world reported an increase in the number of studies related to cereal by-products and wastes during the last 20 years [26]. This significant increase seems to be closely related to unified legislation, food safety measures, and CE and SDG requests.

## 3. It Is only Waste if We Waste It!

The growing demand for food, as a result of the increase in the global population, is directly related to the growth in the amount of food waste [27]. Food waste was defined by the Food and Agriculture Organization of the United Nations (2019) according to the following two indices: “Food Loss Index represented by food lost in production or in the supply chain before it reaches the retail level and Food Waste Index regarding food that is subsequently wasted by consumers or retailers”. In total, 14% of the world’s food is lost before it reaches the retail level (Food and Agriculture Organization of the United Nations, 2019). Food losses also include food processing by-products which are produced as side streams during the preparation of final products across all sectors of the food industry [28,29]. 

**Figure 5:**
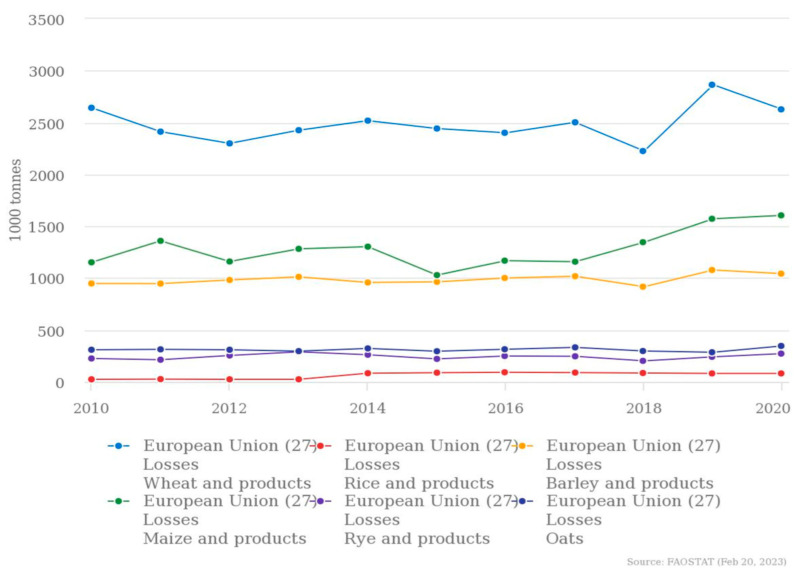
Cereal losses for wheat, maize, rice, rye, barley, oats, and related products in the European Union (27 member states). Source: compiled by authors, based on FAOSTAT online database, 20 February 2023.

Grain processing generates approximately 12.9% of all food waste worldwide [30]. Figure 5 shows the cereal losses in the last decade, according to the FAOSTAT online database, for the main cereals processed in European Union, namely, wheat, maize, rice, rye, barley, and oats, in descending order of quantities.

Although they are rich in nutrients, cereal waste and by-products are mainly used in animal feed and the production of biofuels or are discarded. Nowadays, the grain processing industry is striving not only for the reduction of the volume of waste and by-products but also for the sustainable valorization of the existing ones through the recovery of compounds with added value that are useful in the food industry.

Waste and by-products arise during dry milling (which, mainly, produces flour), wet milling (dedicated mainly to starch and glucose production), and the brewing process. This cereal waste and these by-products may be used for the extraction of bioactive compounds or may be directly used with some modifications for different purposes [30]. Food applications often try to utilize the by-product whole, whereas the strategy within a biorefinery concept targets specific compounds [31].

### 3.1. Conventional Milling, the Major Supplier of Grain Losses Reused in Food Industry

**Figure 6:**
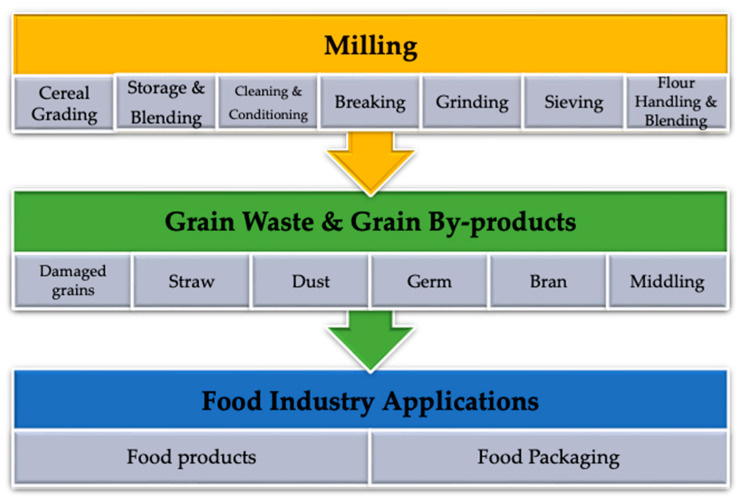
Schematic diagram showing food industry valorization of by-products and waste from dry milling (original source).

The milling process has two ultimate aims: first, to provide quality to the specified product and, second, to efficiently separate the main parts of the grain (bran, germ, and endosperm). The first steps of milling, such as grading, storage, cleaning, and conditioning, are the source of grain waste including damaged grains (shrunken, broken, puny, or sprouted grains), wild plants seeds, substandard grains (predominantly starchy), chaff, remnants of straw and weeds (with a predominance of fiber), and dust. The main stages of cereal dry milling, including breaking, grinding and sieving, produce side streams like germ, bran, and middling. These steps are shown in Figure 6.

Unlike dry milling, wet milling consists of grinding the soaked grains and then separating the chemical compounds of the grains (starch, protein, fiber, and oil) [32]. According to Serna Saldivar [33], industrial-scale wet milling of rye, barley, and oats is very limited or practically nonexistent today because the extraction of starch from rye is difficult due to its higher pentosan content and low gluten-forming capacity; in the same way, wet milling of oats is limited due to difficulties in fully separating the starch because of the hydrated bran and protein layers [32].

In addition to impurities and extraneous matter disposed of in the first steps of the milling process, there are large amounts of dust particles generated during the grain brushing and filtration process. Some studies indicated the feasibility of using organic residues from the milling industry as a fibrous component for biocomposite materials and as a culture medium for microbial cellulose with the aim of developing biopackaging and biodisposable items for the food industry [34]. Desire to reduce the environmental impact of single-use plastic items has led to the investigation of alternatives to fossil-based polymers and the exploration of the opportunities offered by green polymers [35,36]. Tests have been performed with lignocellulosic wastes from milling, such as rice husk, studying their use as fillers for biocomposites used in food packaging and the production of disposable plates [37]. A procedure reported by Torres et al., through which starch was extracted from potato peels (a by-product of food processing), resulted in obtaining a matrix; to reinforce this starch-based matrix obtained from food waste, wheat dust was tested as a filler [38]. The experimental result consisted of biodegradable plate-shaped materials for food use. The use of wheat dust as a cheap raw material for bacterial cellulose culture medium was also explored [34], using a static cultivation method [39]. As a result of the experimental approach, a biofilm was obtained, used as plant-derived cellulose to obtain biodegradable food packaging.

Worldwide, the annual production of wheat bran, obtained as a by-product of grain milling, is about 150 million tons [40], which represents 3–30% of the weight of the kernel in the case of dry milling. By-products of the dry grinding of cereal grain also include hulls, husks (4–14%), germ, broken grains (6–13%), and powders (7–12%) [41]. Of these, the by-product that is most frequently used as a food ingredient is cereal bran, especially in bakery products, and its inclusion is specifically aimed at increasing the dietary fiber content by replacing part of the flour in bread, muffins, shortbread cookies, and cakes [42].

Maize bran as a milling by-product (60–70 g/kg) represents a low-cost source of dietary fiber and natural dietary antioxidants [43]. Often, in the production of bread, high fiber content in the bran and the copresence of lipids and lipase in the germ is considered a disadvantage. Due to the use of fermented by-productst—through the fermentation of lactic bacteria (Lactobacillus plantarum and Weissella confusa)—as ingredients, the nutritional, textural, and sensory properties of wheat bread (containing 25% fermented by-products) have been improved, in the sense of a higher concentration in dietary fiber and proteins (11 and 13% of the dry matter, respectively), a significant increase in protein digestibility (up to 60%), and a consequent decrease in the starch hydrolysis index (13%) [44].

Maize germs from the dry and wet milling processes are used in the food industry for the extraction of edible oil. For the recovery of edible oil from the germ fraction obtained after dry milling corn, mechanical screw presses or a combination of screw presses and solvent extraction are used [32]. In the case of bread fortification with corn germ protein hydrolyzate (1–4%), an improvement in bread texture was demonstrated by reducing hardness and chewiness during storage [45]. By-products rich in fiber, protein, and antioxidants, obtained from the processing of corn starch, can also be added as low-calorie and low-cost agents in food products to partially replace fat or sugar [46]. A recent study concluded that dietary fiber from corn bran can be added to emulsion-based meat products without reducing their sensory and textural quality [11]. Corn bran (5–15 g) added to chicken nuggets improved the texture of the products, in terms of firmness and hardness, in a manner that was directly proportional to the amount of bran added [47]. Another study demonstrated that by replacing lean meat with 3% corn bran, chicken sausages had improved acceptability, higher dietary fiber content, and a longer shelf life [48].

Adding wheat bran to flour is considered a way to improve the nutritional value of bakery products. One of the negative effects of this addition of wheat bran is the fact that a high content of insoluble dietary fibers will affect the quality of the bakery products; namely, their color will be darker after the baking process, the texture will be coarse, and the volume will be reduced, which leads to limitations in its application [49]. Wheat bran slows down the formation of the gluten network if too high a percentage is added. But by adding up to 24% bran content, the dough development time was increased. Another positive consequence of adding wheat bran to flour is the decrease in the electricity consumption required during mixing and in the maximum power consumed, due to the weakening of the gluten structure. Regarding dough rheology, the stickiness and extensibility decreased with an increase in the amount of bran. Wheat bran-supplemented flour demonstrated improved dough aeration during mixing and an improved dough expansion rate during fermentation. On the downside, adding wheat bran decreased the specific volume of bread up to 10.81%, because wheat bran changes the pore size distribution in the crumb [50]. Wheat bran aqueous extracts obtained by ultrasound-assisted technology have been used in pasta making; a study found that enriched pasta had significantly higher antioxidant activity and improved sensory properties when compared to control sample pasta [51]. In the case of meat food products, chicken sausages treated with 6% wheat bran showed a significant increase in gumminess and chewiness; the sensory acceptability of sausages to which 3% fiber was added was comparable to the control sample, but a further increase in fiber level resulted in a decrease in sensory acceptability [52].

Wheat germ is another major by-product of wheat milling. Although it is rich in bioactive components, it has rarely been used in food composition, mainly because of its high lipid content, which makes it subject to rancidity and reduced shelf life [53]. Wheat flour bread was also fortified with 15% fermented milling by-products (using Lactobacillus plantarum and Lactobacillus rossiae), using a dough composed of wheat germ and bran, to obtain a product with 6.53% dietary fiber, or 5% more than in wheat flour bread [54,55]. The glycemic index in vitro and, especially, in vivo was lower for fortified bread, reaching 36.9%, a value far below the threshold required for a food product to be considered as possessing a “low glycemic index” [54]. The addition of wheat germ to bread dough increased water absorption and development time but decreased stability after over-kneading, dough tenacity, extensibility, and dough alveographic strength. Bread made from dough with added wheat germ presented decreased volume, cohesiveness, and elasticity and increased firmness, which could be improved by using certain thermal treatments such as extrusion [56]. Dough rheology and bread properties were also enhanced by adding wheat germ stabilized by heat treatment (toasting) or sourdough fermentation [57]. Studies have been carried out regarding the improvement of dough rheology and bread properties using Chinese steaming, which includes the addition of raw wheat germ or defatted wheat germ. Sensory evaluation ratings and textural analysis indicated that steamed bread with acceptable quality attributes can be prepared with the application of a small amount of wheat germ flour (3–6%) [58,59]. In other studies, a wheat germ level of up to 15% had no significant effect on the sensory characteristics of cookies but improved their nutritional value [60]. Also, the addition of wheat germ in homemade biscuits, at a level of 20% (*w*/*w*), improved the acceptability of sensory characteristics immediately after preparation and during storage and enhanced the nutritional value of the biscuits [61]. The addition of raw and microwaved wheat germ increased cooking losses and the acidity of cooked pasta during storage, and enriched samples were significantly higher in protein, fat, and ash content [62,63,64].

Compared to other cereals, rice is mainly consumed as a whole grain. Therefore, the rice milling industry focuses on reducing the percentage of broken grains so that, apart from the rice bran, there are no significant waste and by-products to be reused in the food industry. Rice is an important food ingredient in Asia; thus, there is also a commercial use for rice bran, the main by-product of rice processing. For example, there is a Japanese dish made from fermented rice bran, “Nukazuke”, which is, basically, a pickled dish prepared from a rice bran bed combined with different vegetables for improved flavor [65]. An important source of edible oil is rice bran, which can represent up to 20% of the oil’s weight [66]. A recent study indicated that substituting stabilized rice bran (after grinding, a stabilization process is required to prevent rancidity) for wheat flour resulted in a significant increase in total antioxidant activity, total dietary fiber content, ashes, and bioactive compounds; however, the results indicated that up to 15% replacement of wheat flour affects the overall physical properties of the dough and the sensory attributes of the bread [67]. Stabilized rice bran improves nutritional value and texture characteristics, thereby promoting the consumption of bakery and pastry products that contribute to a healthy diet, such as biscuits [68,69] and muffins [70]. Another study concluded that the addition of the probiotic *L. casei* strain to rice bran, in yogurt formulations, increased probiotic viability in proportion to the increase in the amount of rice bran (3%); rice bran enrichment resulted in an increase in water-holding capacity and pH and a decrease in syneresis and viscosity values; however, rice bran yogurts had lower sensory scores compared to plain yogurt [71].

Bread containing up to 10% oat bran had acceptable properties [72]. For a higher oat bran percentage, with the intention of improving the quality of bread incorporating 15% oat bran, individual and combined enzymes were used. In this study, to improve the rheological behavior of the dough during the breadmaking process, three enzymes were used: α-amylase, xylanase, and cellulase [73]. A similar study, carried out by Liu et al., stipulated that 15% oat bran be added to wheat flour dough, as the enzyme combination (α-amylase and xylanase) of oat bran can significantly improve the quality of Chinese steamed bread [74]. A recent study reported the effect of adding oat bran to spaghetti pasta dough, from the point of view of cooking quality, product digestibility, and antioxidant, nutritional, and textural characteristics; pasta dough obtained by replacing 50% durum wheat semolina with oat bran resulted in higher cooking losses and higher water absorption index compared to the control sample prepared with 100% durum wheat semolina; also, the caloric content and the digestibility of its starch components were reduced. Thus, this process may represent a healthy option for food diets [75].

Rye is the cereal used predominantly in the diet of the peoples of northern Europe, and it is often used as whole meal flour in the manufacture of bakery products [76]. Rye bran is a by-product of milling and can be used as a valuable additive to increase the nutritional and health properties of food [77].

### 3.2. Brewing Process and Its Wastes

The brewing process generates three intrinsic wastes: brewer’s spent grain (BSG), hot trub, and residual yeast [78,79]. Brewer’s spent grain represents approximately 85% of the total by-products generated by the brewing industry. As the main by-product, brewer’s spent grain is rich in cellulose and non-cellulosic polysaccharides and is the result of the mashing process. This process is one of the early stages of the brewing process and is carried out in the distillery with the aim of solubilizing the malt and cereal grains to ensure proper extraction of the wort (water with the extracted matter) [80].

Brewer’s spent grain consists of husk, pericarp, and seed layers. Residual amounts of endosperm and aleurone from barley are used mainly as a raw material [81]. Due to its properties and because it contains essential nitrogen-containing nutrients, brewer’s spent grain is mainly sold as animal feed, but it has also been shown to have a desirable nutritional value for the human diet [82]. Several researches have shown that brewer’s spent grain can successfully be incorporated into flour used for production of bread, waffles, cookies (with the inclusion of 40% brewer’s spent grain in the flour), breakfast cereals, pasta, pancakes, or tortillas; the obtained results have revealed that the addition of brewer’s spent grain to wheat flour bread increases the amount of fiber, changes the fat content of the product, increases the water holding capacity and texture of the products, and gives the product a slightly sweeter taste [83,84,85,86,87]. The addition of brewer’s spent grain does not adversely affect the sensory characteristics and physicochemical quality indicators of meat products; moreover, it enhances the health-promoting properties of food such as meat sausages [88,89].

Hot trub is another brewing process by-product, represented by sediments formed in the brewing process during the boiling of the wort, and is the least-used by-product in the food industry due to the bitterness that comes from its ingredients [82]. There are studies focused on the development of an extraction process that leads to the reduction of the bitter taste without changing the characteristics (it can even improve them); as a result, hot trub with modified composition and functionality can be used in the food industry to enrich high-fat products or as an alternative source of plant-based proteins [90].

The use of the third brewery by-product, spent yeast, is reduced due to the presence of hops in the boiled wort, which imparts a strong, bitter taste, although there are methods to mitigate this taste [90]. There are also studies about food products with brewer’s spent yeast used as food additive [91]. This by-product has also been used in addition-fortified vegan cakes, resulting in higher vegetable protein, lipid, and carbohydrate content [92]. According to another study, experiments were performed in which spent dry yeast was added as an ingredient to homemade bread. This resulted in an increase in β-glucan intake [93].

## 4. Added-Value Compounds for Food Industry

**Figure 7:**
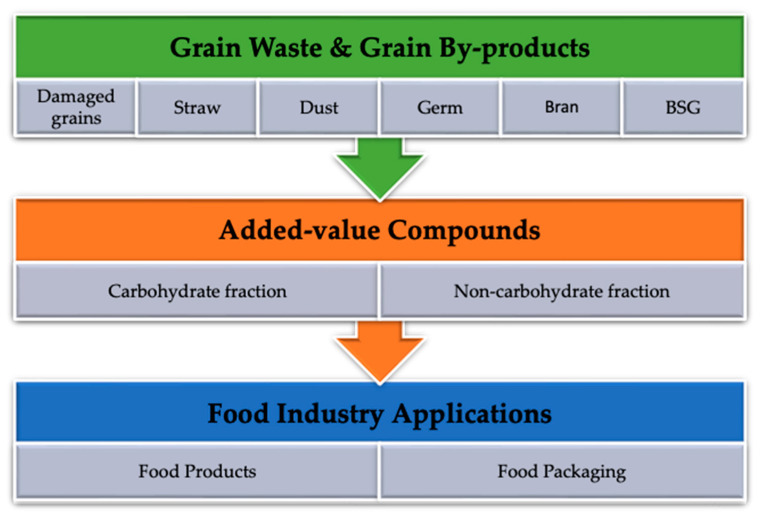
Schematic diagram showing food industry applications of added-value compounds from grain waste and grain by-products (original source).

Grain processing wastes and by-products can not only be directly incorporated into food products but can also be used for the extraction of value-added compounds that can be introduced into the food industry production process as functional food ingredients, as shown in Figure 7. 

The treatment of cereal industrial wastes uses physicochemical and biological methods at high conversion costs [20,94,95]. Therefore, the extraction and use of these valuable compounds are less frequent on an industrial scale in the food industry and more frequent in biorefineries for conversion into fuel as a renewable source of energy. Currently, cereal by-products have been linked to health promotion due to their rich content of fiber, minerals, vitamins, phenolic compounds, phytosterols, policosanols, and other phytochemicals responsible for reducing oxidative stress and mediating the inflammatory process and excretion and absorption of lipids [42]. For this reason, researches related to the recovery of the biological compounds from grain processing waste and by-products also address to the benefits brought to the improvement of products in the food industry.

### 4.1. Carbohydrate Fraction 

The grain processing chain generates significant amounts of waste known as lignocellulosic biomass [14,96]. Grain wastes that include carbohydrate fractions, especially hemicelluloses, are bran, straw, and hulls/husks. 

Of total agricultural waste, 20–35% is hemicellulose, a dietary fiber that represents the most promising source for valuable applications [97]. Industrial-scale applications of hemicelluloses are still underutilized at this point [98]. Hemicellulose demonstrates excellent properties, including biodegradability, biocompatibility, and bioactivity, which also enable it to be applied in the food industry [99], as presented in Table 2. 

Beta-glucans and arabinoxylans from cereals make them valuable components of dietary fibers. Because of high processing costs, there is a limited use of pure preparations of beta-glucans as a food ingredient; instead, the use of bulk fractions and the use of novel separation and purification processes may bring effective solutions [29]. The extraction of hemicelluloses (such as arabinoxylans and β-glucans) from the cell walls and from fractionation and purification can be used in various food applications. The extraction process is possible through four main types of methods: water extraction, which can be carried out at low or high temperatures, chemical extraction (with acids, alkalis, or organic solvents), specific enzymes’ extraction, or mechanical treatments (microwave, ultrasound, extrusion) [97,100]. Herrera-Balandrano et al. demonstrated that it is possible to add impure hemicellulose fractions to food products to improve sensory and chemical properties; for example, according to a study, the addition of 0.15% and 0.30% nixtamalized corn bran can increase the antioxidant capacity, phenolic content, and physicochemical properties of Frankfurter sausages [101].

**Table 2:** Applications of carbohydrate fractions from grain by-products and waste in the food industry.

Grain By-Product/Waste	Carbohydrate Fraction	Food Industry Applications	Source
Wheat bran Rye bran BSG *	Cellulose	Improving sensory and chemical properties of food products	[26,101,102,103,104,105,106]
Cereal bran	Arabinoxylans	Packaging materials (films) Thickening and stabilizing agent in the food industry	[102,106,107,108,109]
Wheat bran Corn bran BSG *	Lignin	Emulsifying stability; dispersing and binding agent	[26,102,103,106,109,110,111]
Oat bran BSG *	Beta-glucans	Wheat flour substitutes Improve beverage satiety Food hydrocolloids	[26,102,112,113]
BSG *	Residual undigested starch	Prebiotic ingredients for the meat industry	[26,112,114,115]
Wheat bran	Lactic and succinic acids	Acidulant, flavoring, preservative agent in the food industry	[116]
Wheat germ	Linoleic acid,	Food ingredient with potential health benefits	[117,118,119]
Corn germ	palmitic acid,	Commercial shortening replacement in food industries	
Rye bran	oleic acid	Food-grade ingredient	
Wheat germ Rye bran	Linolenic acid	Food-grade ingredient	[117,119]
Corn germ	Stearic acid	Commercial shortening replacement in food industries	[118]

* BSG—brewer’s spent grain.

Organic acid (linoleic, linolenic, palmitic, oleic, stearic, lactic, and succinic acids), another carbohydrate fraction, is obtained by fermentative production and is used as an acidulant, flavoring agent, or preservative in the food industry, as depicted in Table 2. 

Carbohydrate fractions from cereal processing by-products and waste can also be an important source for the food packaging industry due to the lignocellulosic materials that they contain, which can be utilized as low-cost substrates for the production of PHA-polyhydroxyalkanoates [94] and PHB-poly-3-hyrdroxybutyrate (Table 3). Enzymatic actions transform the lignocellulosic material in fermentable sugars and then the material is fermented by different bacteria (Enterococcus, Lactobacillus, Leuconostoc, and Streptococcus) and fungi (Rhizopus Monilina and Mucor) [94,120]. The first report of biopolymer production from mild acid-pretreated rice straw (using Bacillus firmus NII 0830) [121] emphasized the possibility of the replacement of petrochemical-derived plastics by the biopolymer poly-3-hydroxybutyrate (PHB). Maximum PHB production was 1.697 g/L from 1.9 g/L biomass; the highest value (89% of biomass) was reported from *Bacillus* species. And yet, the high operational cost of this PHA is a big disadvantage in industrial production and commercialization [94,122].

### 4.2. Non-Carbohydrate Fraction

The food industry mainly exploits the non-carbohydrate fraction represented by proteins and phenolic compounds extracted from grain processing waste and by-products (Table 4).

The protein extracted from corn by-products has a unique structure, molecular shape, and solubility, forming a uniform, transparent, and soft film with good oil and water retention characteristics that make it useful in food preservation [12]. One of the best potential sources of vegetable protein for the food industry is brewer’s spent grain, due to its high protein content, which represents about 20% in dry matter [142]. The food industry can benefit from the use of protein hydrolysates as texture improvers and food additives [129]. Considering the importance of these added-value compounds for the food industry, many studies have concentrated on efficient extraction techniques such as alkaline extraction [132], enzyme-assisted extraction [134], microwave-assisted enzymatic extraction [135,143], and sequential aqueous and alkaline (110 mM NaOH) extraction followed by isoelectric precipitation (pH 3.8), [136] and sodium hydroxide (110 mM) and ultrasound treatment (power 250 W, duty cycle 60%, 20 min/25 °C) [137].

Phenolic compounds can be found mainly in bran, so after its separation from the grain, an extraction process is required. The extraction of polyphenols from cereal by-products can be carried out with various techniques: acid and alkaline hydrolysis [144,145], ultrasound assisted extraction [89,146] microwave-assisted extraction [147], extraction with supercritical carbon dioxide [148], extraction by steam explosion treatment of grain by-products [149], and enzymatic hydrolysis [150]. Wheat and oat bran contain the main phenolic compounds, represented by phenolic acids (ferulic acid, caffeic acid, vanillic acid, p-coumaric acid, dihydroxybenzoic acid, and avenanthramide) and flavonoid subclasses [151]. In rye bran, the most important phenolic compounds (for antioxidant activity) include the group represented by p-hydroxybenzoic acid and its derivatives (especially vanillic and syringic acid) and the group represented by p-coumaric acid and its derivatives (ferulic and caffeic acid) [152]. Phenolic acids, as antioxidant compounds recovered from grain waste and by-products, are used in the food industry as additives to extend the shelf life of food [96]. Vanillin from ferulic acid through biotechnical processes is very often used as a flavoring in the food industry [116].

The use of rice bran proteins and of phenolic and aromatic compounds from wheat bran and germ has been studied [26,153,154,155] and represents an example of the application in food packaging of the non-carbohydrate fraction of grain by-products/waste (Table 5).

## 5. Conclusions and Future Discussion

To sustain the livelihoods of current and future generations, sustainable food consumption and production is an important target of circular economy and of the Sustainable Development Goals. For this reason, upcycling of food processing by-products and waste to secure the food supply chain of an increasing world population—and because of the inevitable diminishing of fossil resources—must be a primary duty of humanity.

The large volume of low-cost by-products and waste from grain processing provides the economical advantage of its potentially valuable components for the food industry. The present paper, carried out using a review of the literature concerning grain waste and by-product recovery and their industrial applications in the food industry, shows that the majority of researches focus on restricted examples and pilot-scale laboratory experiences, which are currently too costly to upscale, while only a few cases study existing full-scale examples. The main application of these processes on an industrial scale consists of the direct incorporation of grain by-product waste with classic ingredients from the bakery, meat, and dairy industries in order to improve the nutritional, rheological, or sensory properties of the final product. The results highlight the use of grain waste and by-products mainly as additives, for texture improvement, as acidulant, flavoring agent, or preservative in food production, or as biodegradable materials (e.g., paper and biofilm for food packaging).

More insights and more in-depth investigations are needed to explore the applications that involve the added-value compounds from grain by-products and waste to produce food; there is an imperative need to develop new and innovative technologies for efficient treatment and extraction, on an industrial scale, and to fulfill the “zero-waste economy” principles.

Few specific case studies in the field of grain processing are related to logistic concerns of industrial symbiosis, for example, the quantitative and qualitative indicators of grain processing waste and by-products obtained by a company, the geographical distribution of possible beneficiaries of those grain losses, unified worldwide regulatory re-strictions, safety concerns, and well-established logistics associated with grain waste collection, transport, and handling. Improving the efficiency of the food value chain could also help bring down the cost of food products intended for human consumption.

## Figures and Tables

**Table 1:** Top 16 most-productive journals. Data compiled by authors.

Journal ^1^	Documents/Year	Citation
*Waste and Biomass Valorization*	18	200
*Journal of Cleaner Production*	15	410
*Bioresource Technology*	9	1800
*Waste Management*	8	172
*Sustainability*	8	59
*Animals*	8	133
*Foods*	7	57
*Applied Sciences—Basel*	7	48
*Food Chemistry*	6	273
*Trends in Food Science and Technology*	6	312
*Molecules*	6	49
*Journal of the Institute of Brewing*	5	338
*Process Biochemistry*	5	203
*Animal Feed Science and Technology*	5	220
*Materials*	5	30
*Resources Conservation and Recycling*	5	166

Notes: ^1^ ranking for the last decade.

**Table 3:** Applications of carbohydrate fractions from grain by-products and waste in food packaging.

Grain By-Product/Waste	Carbohydrate Fraction	Food Packaging Applications	Source
Wheat bran Wheat straw Rice straw Oat husk	Cellulose	PHA *, PHB ** Paper sheet Reinforcing agent for biocomposites in packaging Edible film	[26,121,122,123,124]
Wheat bran	Lactic acid	Packaging, films, and edible coatings with PLA ***	[26,125,126,127,128]

* PHA-polyhydroxyalkanoates, ** PHB-poly-3-hydroxybutyrate (biodegradable polymer film), *** PLA-polylactic acid.

**Table 4:** Applications of non-carbohydrate fraction from grain by-products/waste in the food industry.

Grain By-Product/Waste	Non-Carbohydrate Fraction	Food Industry Applications	Source
BSG * Wheat bran concentrate Wheat germ (raw, defatted, thermally treated) Defatted corn germ Defatted oat bran Defatted Rice bran Malted barley germs	Protein	Texture improvers and food additives Enriching food products showed excellent functional properties in terms of high solubility, good water, and fat absorption capacity Good vegetable protein supplement for cereal-based diets Strong antioxidant activity in food	[29,60,61,62,63,64,129,130,131,132,133,134,135,136,137,138,139,140]
Cereal (wheat, rice, oat) bran	Phenolic compounds	Functional food ingredient Additives to extend the shelf life of food Flavoring	[96,116,141]

* BSG—brewer’s spent grain.

**Table 5:** Applications in food packaging of non-carbohydrate fraction from grain by-products/waste.

Grain By-Product/Waste	Non-Carbohydrate Fraction	Food Packaging Applications	Source
Rice bran	Protein	Component in biodegradable film	[26,153,154]
Wheat bran Wheat germ	Phenolic and aromatic compounds	Production of natural polyesters	[26,155]

## Data Availability

The data used to support the findings of this study can be made available by the corresponding author upon request.

## References

[1] Bhatia L., Jha H., Sarkar T., Sarangi P.K. (2023). Food Waste Utilization for Reducing Carbon Footprints towards Sustainable and Cleaner Environment: A Review. Int. J. Environ. Res. Public Health.

[2] Liu F., Li M., Wang Q., Yan J., Han S., Ma C., Ma P., Liu X., McClements D.J. (2022). Future foods: Alternative proteins, food architecture, sustainable packaging, and precision nutrition. Crit. Rev. Food Sci. Nutr..

[3] Nenciu F., Voicea I., Cocarta D.M., Vladut V.N., Matache M.G., Arsenoaia V.-N. (2022). “Zero-Waste” Food Production System Supporting the Synergic Interaction between Aquaculture and Horticulture. Sustainability.

[4] Gautam M., Agrawal M. (2021). Greenhouse Gas Emissions from Municipal Solid Waste Management: A Review of Global Scenario. Carbon Footprint Case Studies.

[5] Andreottola G., Ragazzi M., Foladori P., Villa R., Langone M., Rada E.C. (2012). The unit intregrated approch for OFMSW treatment. UPB Sci. Bull. Ser. C Electr. Eng..

[6] Cocarta D.M., Rada E.C., Ragazzi M., Badea A., Apostol T. (2009). A contribution for a correct vision of health impact from municipal solid waste treatments. Environ. Technol..

[7] Poole N., Donovan J., Erenstein O. (2021). Viewpoint: Agri-Nutrition Research: Revisiting the Contribution of Maize and Wheat to Human Nutrition and Health. Food Policy.

[8] Global and Regional Food Consumption Patterns and Trends. https://www.fao.org/3/ac911e/ac911e05.htm.

[9] Guerrini A., Burlini I., Huerta Lorenzo B., Grandini A., Vertuani S., Tacchini M., Sacchetti G. (2020). Antioxidant and Antimicrobial Extracts Obtained from Agricultural By-Products: Strategies for a Sustainable Recovery and Future Perspectives. Food Bioprod. Process..

[10] Shiferaw B., Smale M., Braun H.J., Duveiller E., Reynolds M., Muricho G. (2013). Crops That Feed the World 10. Past Successes and Future Challenges to the Role Played by Wheat in Global Food Security. Food Sec..

[11] Fukagawa N.K., Ziska L.H. (2019). Rice: Importance for Global Nutrition. J. Nutr. Sci. Vitaminol..

[12] Zhang R., Ma S., Li L., Zhang M., Tian S., Wang D., Liu K., Liu H., Zhu W., Wang X. (2021). Comprehensive Utilization of Corn Starch Processing By-Products: A Review. Grain Oil Sci. Technol..

[13] Food and Agriculture Organization of the United Nations FAO Cereal Supply and Demand Brief World Food Situation. https://www.fao.org/worldfoodsituation/csdb/en/.

[14] Ouro-Salim O., Guarnieri P. (2022). Circular economy of food waste: A literature review. Environ Qual. Manag..

[15] Teigiserova D.A., Bourgine J., Thomsen M. (2021). Closing the Loop of Cereal Waste and Residues with Sustainable Technologies: An Overview of Enzyme Production via Fungal Solid-State Fermentation. Sustain. Prod. Consum..

[16] Palermito F., Magaril E., Conti F., Kiselev A., Rada E.C. (2021). Circular economy concepts applied to waste anaerobic digestion plants. WIT Transact. Ecol. Environ..

[17] Pakseresht A., Ahmadi Kaliji S., Xhakollari V. (2022). How Blockchain Facilitates the Transition toward Circular Economy in the Food Chain?. Sustainability.

[18] Shelepina N.V., Gorina L.N. (2022). Scientific rationale for reducing the negative impact on the environment of grain processing industries through the rational use of secondary raw materials. IOP Conf. Series: Earth Environ Sci..

[19] Rehal J., Kaur K., Kaur P. (2023). Cereal Grains: Composition, Nutritional Attributes, and Potential Applications. Cereals and Their By-Products.

[20] Kliopova I., Staniškis J.K., Petraškiene V. (2013). Solid recovered fuel production from biodegradable waste in grain processing industry. Waste Manag. Res..

[21] Jain S., Gualandris J. (2023). When does upcycling mitigate climate change? The case of wet spent grains and fruit and vegetable residues in Canada. J. Ind. Ecol..

[22] Zabaniotou A., Kamaterou P. (2019). Food Waste Valorization Advocating Circular Bioeconomy—A Critical Review of Potentialities and Perspectives of Spent Coffee Grounds Biorefinery. J. Clean. Prod..

[23] Thürer M., Tomašević I., Stevenson M., Qu T., Huisingh D. (2018). A Systematic Review of the Literature on Integrating Sustain-ability into Engineering Curricula. J. Clean. Prod..

[24] Gutiérrez-Salcedo M., Martínez M.Á., Moral-Munoz J.A., Herrera-Viedma E., Cobo M.J. (2018). Some Bibliometric Procedures for Analyzing and Evaluating Research Fields. Appl. Intell..

[25] Jan van Eck N., Waltman L. (2023). VOSviewer Manual. https://www.vosviewer.com/.

[26] Skendi A., Zinoviadou K.G., Papageorgiou M., Rocha J.M. (2020). Advances on the Valorisation and Functionalization of By-Products and Wastes from Cereal-Based Processing Industry. Foods.

[27] Belc N., Mustatea G., Apostol L., Iorga S., Vlăduț V.-N., Mosoiu C. (2019). Cereal Supply Chain Waste in the Context of Circular Economy. E3S Web Conf..

[28] (2022). Encyclopedia, Processing of Cereals and Derived-By-Products. https://encyclopedia.pub/entry/2020.

[29] Roth M., Jekle M., Becker T. (2019). Opportunities for Upcycling Cereal Byproducts with Special Focus on Distiller’s Grains. Trends Food Sci. Technol..

[30] Fărcaș A.C., Socaci S.A., Nemeș S.A., Pop O.L., Coldea T.E., Fogarasi M., Biriș-Dorhoi E.S. (2022). An Update Regarding the Bioactive Compound of Cereal By-Products: Health Benefits and Potential Applications. Nutrients.

[31] Tufail T., Ain H.B.U., Saeed F., Nasir M., Basharat S., Mahwish, Rusu A.V., Hussain M., Rocha J.M., Trif M. (2022). A Ret-rospective on the Innovative Sustainable Valorization of Cereal Bran in the Context of Circular Bioeconomy Innovations. Sustainability.

[32] Papageorgiou M., Skendi A. (2018). Introduction to Cereal Processing and By-Products. Sustainable Recovery and Reutilization of Cereal Processing By-Products.

[33] Serna-Saldivar S.O. (2012). Wet-Milling Processes and Starch Properties and Characteristics. Cereal Grains.

[34] Comino E., Dominici L., Perozzi D. (2021). Do-It-Yourself Approach Applied to the Valorisation of a Wheat Milling Industry’s by-Product for Producing Bio-Based Material. J. Clean. Prod..

[35] Ahmadzadeh S., Khaneghah A.M. (2020). Role of Green Polymers in Food Packaging. Encyclopedia of Renewable and Sustainable Materials.

[36] Jin T.Z., Liu L. (2020). Roles of Green Polymer Materials in Active Packaging. ACS Symposium Series.

[37] Sánchez-Safont E.L., Aldureid A., Lagarón J.M., Gámez-Pérez J., Cabedo L. (2018). Biocomposites of Different Lignocellulosic Wastes for Sustainable Food Packaging Applications. Compos. B Eng..

[38] Torres M.D., Fradinho P., Rodríguez P., Falqué E., Santos V., Domínguez H. (2020). Biorefinery Concept for Discarded Potatoes: Recovery of Starch and Bioactive Compounds. J. Food Eng..

[39] Azeredo H.M.C., Barud H., Farinas C.S., Vasconcellos V.M., Claro A.M. (2019). Bacterial Cellulose as a Raw Material for Food and Food Packaging Applications. Front Sustain. Food Syst..

[40] Duţă D.E., Culeţu A., Mohan G. (2018). Reutilization of Cereal Processing By-Products in Bread Making. Sustainable Recovery and Reutilization of Cereal Processing By-Products.

[41] Salazar-López N.J., Ovando-Martínez M., Domínguez-Avila J.A. (2020). Cereal/Grain By-products. Food Wastes By-Products.

[42] Melini V., Melini F., Luziatelli F., Ruzzi M. (2020). Functional Ingredients from Agri-Food Waste: Effect of Inclusion Thereof on Phenolic Compound Content and Bioaccessibility in Bakery Products. Antioxidants.

[43] Hussain M., Qamar A., Saeed F., Rasheed R., Niaz B., Afzaal M., Mushtaq Z., Anjum F. (2021). Biochemical properties of maize bran with special reference to different phenolic acids. Int. J. Food. Prop..

[44] Pontonio E., Dingeo C., Gobbetti M., Rizzello C.G. (2019). Maize Milling By-Products: From Food Wastes to Functional Ingredients through Lactic Acid Bacteria Fermentation. Front. Microbiol..

[45] Karimi A., Gavlighi H.A., Sarteshnizi R.A., Udenigwe C.C. (2021). Effect of Maize Germ Protein Hydrolysate Addition on Digestion, in Vitro Antioxidant Activity and Quality Characteristics of Bread. J. Cereal Sci..

[46] Grasso S. (2020). Extruded Snacks from Industrial By-Products: A Review. Trends Food Sci. Technol..

[47] Pathera A.K., Riar C.S., Yadav S., Singh P.K. (2018). Effect of Egg Albumen, Vegetable Oil, Corn Bran, and Cooking Methods on Quality Characteristics of Chicken Nuggets Using Response Surface Methodology. Food Sci. Anim. Resour..

[48] Yadav S., Malik A., Pathera A., Islam R.U., Sharma D. (2016). Development of Dietary Fibre Enriched Chicken Sausages by In-corporating Corn Bran, Dried Apple Pomace and Dried Tomato Pomace. Nutr. Food Sci..

[49] Yan J., Lv Y., Ma S. (2022). Wheat Bran Enrichment for Flour Products: Challenges and Solutions. J. Food Process Preserv..

[50] Packkia-Doss P.P., Chevallier S., Pare A., Le-Bail A. (2019). Effect of Supplementation of Wheat Bran on Dough Aeration and Final Bread Volume. J. Food Eng..

[51] Pasqualone A., Delvecchio L.N., Gambacorta G., Laddomada B., Urso V., Mazzaglia A., Ruisi P., Di Miceli G. (2015). Effect of Supplementation with Wheat Bran Aqueous Extracts Obtained by Ultrasound-Assisted Technologies on the Sensory Properties and the Antioxidant Activity of Dry Pasta. Natural Prod. Commun..

[52] Yadav S., Pathera A.K., Islam R.U., Malik A.K., Sharma D.P. (2018). Effect of Wheat Bran and Dried Carrot Pomace Addition on Quality Characteristics of Chicken Sausage. Asian-Australas J. Anim. Sci..

[53] Abu-Ghannam N., Balboa E. (2018). Biotechnological, Food, and Health Care Applications. https://arrow.tudublin.ie/schfsehbk/21/253-278.

[54] Pontonio E., Lorusso A., Gobbetti M., Rizzello C.G. (2017). Use of Fermented Milling By-Products as Functional Ingredient to Develop a Low-Glycaemic Index Bread. J. Cereal Sci..

[55] Schiavon M., Ragazzi M., Rada E.C. (2013). A proposal for a diet-based local PCDD/F deposition limit. Chemosphere.

[56] Gomez M., Gonzales J., Oliete B. (2012). Effect of extruded wheat germ on dough rheology and bread quality. Food Bioprocess Technol..

[57] Marti A., Torri L., Casiraghi M.C., Franzetti L., Limbo S., Morandin F., Quaglia L., Pagani M.A. (2014). Wheat germ stabilization by heat-treatment or sourdough fermentation: Effects on dough rheology and bread properties. Lebensm. Wiss. Und Technol. Food Sci. Technol..

[58] Sun R., Zhang Z., Hu X., Xing Q., Zhuo W. (2015). Effect of wheat germ flour addition on wheat flour, dough and Chinese steamed bread properties. J. Cereal Sci..

[59] Ma S., Wang X.X., Zheng X.L., Tian S.Q., Liu C., Li L., Ding Y.F. (2014). Improvement of the quality of steamed bread by supplementation of wheat germ from milling process. J. Cereal Sci..

[60] Petrović J., Rakić D., Fišteš A., Pajin B., Lončarević I., Tomović V., Zarić D. (2017). Defatted wheat germ application: Influence on cookies’ properties with regard to its particle size and dough moisture content. Food Sci. Technol. Int..

[61] Al-Marazeeq K.M., Angor M.M. (2017). Chemical characteristic and sensory evaluation of biscuit enriched with wheat germ and the effect of storage time on the sensory properties for this product. Food Nutr. Sci..

[62] Pınarlı İ., İbanoğlu Ş., Öner M.D. (2004). Effect of storage on the selected properties of macaroni enriched with wheat germ. J. Food Eng..

[63] Tarzi B.G., Shakeri V., Ghavami M. (2012). Quality evaluation of pasta enriched with heated and unheated wheat germ during storage. Adv. Environ. Biol..

[64] Aktaş K., Bilgiçli N., Levent H. (2015). Influence of wheat germ and β-glucan on some chemical and sensory properties of Turkish noodle. J. Food Sci. Technol..

[65] Spaggiari M., Dall’asta C., Galaverna G., Bilbao M.D.D.C. (2021). Rice Bran By-Product: From Valorization Strategies to Nutritional Perspectives. Foods.

[66] Patel M., Naik S.N. (2004). Gamma-Oryzanol from Rice Bran Oil—A Review. https://www.researchgate.net/publication/239785419_Gamma-Oryzanol_from_rice_bran_oil-A_review.

[67] Espinales C., Cuesta A., Tapia J., Palacios-Ponce S., Peñas E., Martínez-Villaluenga C., Espinoza A., Cáceres P.J. (2022). The Effect of Stabilized Rice Bran Addition on Physicochemical, Sensory, and Techno-Functional Properties of Bread. Foods.

[68] de Souza C.B., Lima G.P.P., Borges C.V., Dias L.C.G.D., Spoto M.H.F., Castro G.R., Corrêa C.R., Minatel I.O. (2019). Development of a Functional Rice Bran Cookie Rich in γ-Oryzanol. J. Food Meas. Charact..

[69] Bultum L.E., Emire S.A., Wolde Y.T. (2020). Influence of Full Fat Rice Bran from Ethiopian Rice Milling Industries on Nutritional Qualities, Physicochemical and Sensory Properties of Bread and Biscuits. J. Food Meas. Charact..

[70] Kaur A., Virdi A.S., Singh N., Singh A., Kaler R.S.S. (2021). Effect of Degree of Milling and Defatting on Proximate Composition, Functional and Texture Characteristics of Gluten-Free Muffin of Bran of Long-Grain Indica Rice Cultivars. Food Chem..

[71] Demirci T., Aktaş K., Sözeri D., Öztürk H.İ., Akın N. (2017). Rice Bran Improve Probiotic Viability in Yoghurt and Provide Added Antioxidative Benefits. J. Funct. Foods.

[72] Saka M., Özkaya B., Saka İ. (2021). The Effect of Bread-Making Methods on Functional and Quality Characteristics of Oat Bran Blended Bread. Int. J. Gastron. Food Sci..

[73] Liu W., Brennan M., Tu D., Brennan C. (2023). Influence of α-Amylase, Xylanase and Cellulase on the Rheological Properties of Bread Dough Enriched with Oat Bran. Sci. Rep..

[74] Liu W., Brennan M., Brennan C., You L., Tu D. (2023). Effect of Enyzmes on the Quality and Predicting Glycaemic Response of Chinese Steamed Bread. Foods.

[75] Espinosa-Solis V., Zamudio-Flores P.B., Tirado-Gallegos J.M., Ramírez-Mancinas S., Olivas-Orozco G.I., Espino-Díaz M., Hernández-González M., García-Cano V.G., Sánchez-Ortíz O., Buenrostro-Figueroa J.J. (2019). Evaluation of Cooking Quality, Nutritional and Texture Characteristics of Pasta Added with Oat Bran and Apple Flour. Foods.

[76] Verni M., Rizzello C.G., Coda R. (2019). Fermentation Biotechnology Applied to Cereal Industry By-Products: Nutritional and Functional Insights. Front. Nutr..

[77] Dziki D. (2022). Rye Flour and Rye Bran: New Perspectives for Use. Process.

[78] Ortiz I., Torreiro Y., Molina G., Maroño M., Sánchez J.M. (2019). A Feasible Application of Circular Economy: Spent Grain Energy Recovery in the Beer Industry. Waste Biomass Valoriz.

[79] dos Santos Mathias T.R., Alexandre V.M.F., Cammarota M.C., de Mello P.P.M., Sérvulo E.F.C. (2015). Characterization and de-termination of brewer’s solid wastes composition. J. Inst. Brew..

[80] Bharat Helkar P., Sahoo A., Patil N. (2016). Review: Food Industry By-Products Used as a Functional Food Ingredients. Int. J. Waste Resour..

[81] Chetrariu A., Dabija A. (2020). Brewer’s Spent Grains: Possibilities of Valorization, a Review. Appl. Sci..

[82] Rachwał K., Waśko A., Gustaw K., Polak-Berecka M. (2020). Utilization of Brewery Wastes in Food Industry. PeerJ.

[83] Cappa C., Cappa C. (2017). Brewer’s Spent Grain Valorization in Fiber-Enriched Fresh Egg Pasta Production: Modelling and Optimization Study. Food Sci. Technol..

[84] Fărcaş A.C., Socaci S.A., Mudura E., Dulf F.V., Vodnar D.C., Tofană M., Salanță L.C., Fărcaş A.C., Socaci S.A., Mudura E. (2017). Exploitation of Brewing Industry Wastes to Produce Functional Ingredients. Brewing Technology.

[85] Combest S., Warren C. (2019). Perceptions of College Students in Consuming Whole Grain Foods Made with Brewers’ Spent Grain. Food Sci. Nutr..

[86] Ktenioudaki A., Chaurin V., Reis S.F., Gallagher E. (2012). Brewer’s Spent Grain as a Functional Ingredient for Breadsticks. Int. J. Food Sci. Technol..

[87] Ktenioudaki A., O’Shea N., Gallagher E. (2013). Rheological Properties of Wheat Dough Supplemented with Functional By-Products of Food Processing: Brewer’s Spent Grain and Apple Pomace. J. Food Eng..

[88] Nagy M., Semeniuc C.A., Socaci S.A., Pop C.R., Rotar A.M., Sălăgean C.D., Tofană M. (2017). Utilization of Brewer’s Spent Grain and Mushrooms in Fortification of Smoked Sausages. Food Sci. Technol..

[89] Choi M.S., Choi Y.S., Kim H.W., Hwang K.E., Song D.H., Lee S.Y., Kim C.J. (2014). Effects of Replacing Pork Back Fat with Brewer’s Spent Grain Dietary Fiber on Quality Characteristics of Reduced-Fat Chicken Sausages. Food Sci. Anim. Resour..

[90] Saraiva B.R., Anjo F.A., Vital A.C.P., da Silva L.H.M., Ogawa C.Y.L., Sato F., Coimbra L.B., Matumoto-Pintro P.T. (2019). Waste from Brewing (Trub) as a Source of Protein for the Food Industry. Int. J. Food Sci. Technol..

[91] Naibaho J., Korzeniowska M. (2021). Brewers’ spent grain in food systems: Processing and final products quality as a function of fiber modification treatment. J. Food Sci..

[92] Coldea T.E., Mudura E., Rotar A.M., Cuibus L., Pop C.R., Darab C. (2017). Brewer’s Spent Yeast Exploitation in Food Industry. Hop Med. Plants.

[93] Martins Z.E., Erben M., Gallardo A.E., Silva R., Barbosa I., Pinho O., Ferreira I.M.P.L.V.O. (2015). Effect of Spent Yeast Fortification on Physical Parameters, Volatiles and Sensorial Characteristics of Home-Made Bread. Int. J. Food. Sci. Technol..

[94] Hassan G., Shabbir M.A., Ahmad F., Pasha I., Aslam N., Ahmad T., Rehman A., Manzoor M.F., Inam-Ur-Raheem M., Aadil R.M. (2021). Cereal Processing Waste, an Environmental Impact and Value Addition Perspectives: A Comprehensive Treatise. Food Chem..

[95] Rada E.C., Ragazzi M., Fiori L., Antolini D. (2009). Bio-drying of grape marc and other biomass: A comparison. Water Sci. Technol..

[96] Fărcaș A.C., Socaci S.A., Nemeș S.A., Salanță L.C., Chiș M.S., Pop C.R., Borșa A., Diaconeasa Z., Vodnar D.C. (2022). Cereal Waste Valorization through Conventional and Current Extraction Techniques—An Up-to-Date Overview. Foods.

[97] Arzami A.N., Ho T.M., Mikkonen K.S. (2022). Valorization of Cereal By-Product Hemicelluloses: Fractionation and Purity Con-siderations. Food Res. Int..

[98] Qaseem M.F., Shaheen H., Wu A.M. (2021). Cell Wall Hemicellulose for Sustainable Industrial Utilization. Renew Sustain. Energ. Rev..

[99] Luo Y., Li Z., Li X., Liu X., Fan J., Clark J.H., Hu C. (2019). The Production of Furfural Directly from Hemicellulose in Lignocel-lulosic Biomass: A Review. Catal. Today.

[100] Valoppi F., Wang Y.J., Alt G., Peltonen L.J., Mikkonen K.S. (2021). Valorization of Native Soluble and Insoluble Oat Side Streams for Stable Suspensions and Emulsions. Food Bioproc. Technol..

[101] Herrera-Balandrano D.D., Báez-González J.G., Carvajal-Millán E., Méndez-Zamora G., Urías-Orona V., Amaya-Guerra C.A., Niño-Medina G. (2019). Feruloylated Arabinoxylans from Nixtamalized Maize Bran Byproduct: A Functional Ingredient in Frankfurter Sausages. Molecules.

[102] Dapčević-Hadnađev T., Hadnađev M., Pojić M., Galanakis C.M. (2018). 2-The healthy components of cereal by-products and their functional properties. Sustainable Recovery and Reutilization of Cereal Processing by-Products.

[103] Ciudad-Mulero M., Fernández-Ruiz V., Matallana-González M.C., Morales P., Ferreira I.C.F.R., Barros L. (2019). Chapter Two—Dietary Fiber Sources and Human Benefits: The Case Study of Cereal and Pseudocereals. Advances in Food and Nutrition Research.

[104] Juhnevica-Radenkova K., Kviesis J., Moreno D.A., Seglina D., Vallejo F., Valdovska A., Radenkovs V. (2021). Highly-Efficient Release of Ferulic Acid from Agro-Industrial By-Products via Enzymatic Hydrolysis with Cellulose-Degrading Enzymes: Part I–The Superiority of Hydrolytic Enzymes Versus Conventional Hydrolysis. Foods.

[105] Sandak A., Sandak J., Modzelewska I. (2019). Manufacturing fit-for-purpose paper packaging containers with controlled biodegradation rate by optimizing addition of natural fillers. Cellulose.

[106] Ravindran R., Jaiswal A.K. (2016). Exploitation of food industry waste for high-value products. Trends Biotechnol..

[107] Bastos R., Coelho E., Coimbra M.A., Galanakis C.M. (2018). 8-Arabinoxylans from cereal by-products: Insights into structural features, recovery, and applications. Sustainable Recovery and Reutilization of Cereal Processing By-Products.

[108] Pérez-Flores J.G., Contreras-López E., Castañeda-Ovando A., Pérez-Moreno F., Aguilar-Arteaga K., Álvarez-Romero G.A., Téllez-Jurado A. (2019). Physicochemical characterization of an arabinoxylan-rich fraction from brewers’ spent grain and its application as a release matrix for caffeine. Food Res. Int..

[109] Onipe O.O., Jideani A.I.O., Beswa D. (2015). Composition and functionality of wheat bran and its application in some cereal food products. Int. J. Food Sci. Technol..

[110] Gil-Chávez J., Gurikov P., Hu X., Meyer R., Reynolds W., Smirnova I. (2021). Application of novel and technical lignins in food and pharmaceutical industries: Structure-function relationship and current challenges. Biomass Convers. Biorefinery.

[111] Yadav M.P., Parris N., Johnston D.B., Onwulata C.I., Hicks K.B. (2010). Corn fiber gum and milk protein conjugates with improved emulsion stability. Carbohydr. Polym..

[112] Zhu F., Du B., Xu B. (2016). A critical review on production and industrial applications of beta-glucans. Food Hydrocoll..

[113] Izydorczyk M.S., Dexter J.E. (2008). Barley β-glucans and arabinoxylans: Molecular structure, physicochemical properties, and uses in food products—A Review. Food Res. Int..

[114] Parchami M., Ferreira J.A., Taherzadeh M.J. (2021). Starch and protein recovery from brewer’s spent grain using hydrothermal pretreatment and their conversion to edible filamentous fungi—A brewery biorefinery concept. Bioresour. Technol..

[115] Johansson E.V., Nilsson A.C., Östman E.M., Björck I.M.E. (2013). Effects of indigestible carbohydrates in barley on glucose metabolism, appetite and voluntary food intake over 16 h in healthy adults. Nutr. J..

[116] Apprich S., Tirpanalan Ö., Hell J., Reisinger M., Böhmdorfer S., Siebenhandl-Ehn S., Novalin S., Kneifel W. (2014). Wheat Bran-Based Biorefinery 2: Valorization of Products. LWT.

[117] Meriles S.P., Penci M.C., Curet S., Boillereaux L., Ribotta P.D. (2022). Effect of microwave and hot air treatment on enzyme activity, oil fraction quality and antioxidant activity of wheat germ. Food Chem..

[118] Zhao M., Lan Y., Cui L., Monono E., Rao J., Chen B. (2020). Formation, characterization, and potential food application of rice bran wax oleogels: Expeller-pressed corn germ oil versus refined corn oil. Food Chem..

[119] Povilaitis D., Venskutonis P.R. (2015). Optimization of supercritical carbon dioxide extraction of rye bran using response surface methodology and evaluation of extract properties. J. Supercrit. Fluids.

[120] Gholami A., Mohkam M., Rasoul-Amini S., Ghasemi Y. (2016). Industrial production of polyhydroxyalkanoates by bacteria: Opportunities and challenges. Minerva Biotecnol..

[121] Sindhu R., Silviya N., Binod P., Pandey A. (2013). Pentose-rich hydrolysate from acid pretreated rice straw as a carbon source for the production of poly-3-hydroxybutyrate. Biochem. Eng. J..

[122] Obruca S., Benesova P., Marsalek L., Marova I. (2015). Use of lignocellulosic materials for PHA production. Chem. Biochem. Eng. Q..

[123] Bilo F., Pandini S., Sartore L., Depero L.E., Gargiulo G., Bonassi A., Federici S., Bontempi E. (2018). A sustainable bioplastic obtained from rice straw. J. Clean. Prod..

[124] Souza Filho P.F., Zamani A., Ferreira J.A. (2020). Valorization of wheat byproducts for the co-production of packaging material and enzymes. Energies.

[125] Nikkilä M. (2020). Cereal Waste Valorization through Development of Functional Key Fibres to Innovate in Fibre Packaging Materials. http://cordis.europa.eu/project/rcn/110025_en.html.

[126] Lorite G.S., Rocha J.M., Miilumäki N., Saavalainen P., Selkälä T., Morales-Cid G., Gonçalves M.P., Pongrácz E., Rocha C.M.R., Toth G. (2016). Evaluation of physicochemical/microbial properties and life cycle assessment (LCA) of PLA-based nanocomposite active packaging. LWT Food Sci. Technol..

[127] Peelman N., Ragaert P., De Meulenaer B., Adons D., Peeters R., Cardon L., Van Impe F., Devlieghere F. (2013). Application of bioplastics for food packaging. Trends Food Sci. Technol..

[128] Djukić-Vuković A., Mladenović D., Radosavljević M., Kocić-Tanackov S., Pejin J., Mojović L. (2016). Wastes from bioethanol and beer productions as substrates for l(+) lactic acid production—A comparative study. Waste Manag..

[129] Lech M., Labus K. (2022). The Methods of Brewers’ Spent Grain Treatment towards the Recovery of Valuable Ingredients Contained Therein and Comprehensive Management of Its Residues. Chem. Eng. Res. Design.

[130] Alzuwaid N.T., Sissons M., Laddomada B., Fellows C.M. (2020). Nutritional and functional properties of durum wheat bran protein concentrate. Cereal Chem..

[131] Prandi B., Faccini A., Lambertini F., Bencivenni M., Jorba M., Van Droogenbroek B., Bruggeman G., Schöber J., Petrusan J., Elst K. (2019). Food wastes from agrifood industry as possible sources of proteins: A detailed molecular view on the composition of the nitrogen fraction, amino acid profile and racemisation degree of 39 food waste streams. Food Chem..

[132] Zhu K.-X., Zhou H.-M., Qian H.-F. (2006). Proteins Extracted from Defatted Wheat Germ: Nutritional and Structural Properties. Cereal Chem..

[133] Espinosa-Pardo F.A., Savoire R., Subra-Paternault P., Harscoat-Schiavo C. (2020). Oil and protein recovery from corn germ: Extraction yield, composition and protein functionality. Food Bioprod. Process..

[134] Guan X., Yao H. (2008). Optimization of Viscozyme L-assisted extraction of oat bran protein using response surface methodology. Food Chem..

[135] Phongthai S., Lim S.-T., Rawdkuen S. (2016). Optimization of microwave-assisted extraction of rice bran protein and its hydrolysates properties. J. Cereal Sci..

[136] Connolly A., Piggott C.O., FitzGerald R.J. (2013). Characterisation of protein-rich isolates and antioxidative phenolic extracts from pale and black brewers’ spent grain. Int. J. Food Sci. Technol..

[137] Li W., Yang H., Coldea T.E., Zhao H. (2021). Modification of structural and functional characteristics of brewer’s spent grain protein by ultrasound assisted extraction. LWT.

[138] Majzoobi M., Ghiasi F., Farahnaky A. (2016). Physicochemical assessment of fresh chilled dairy dessert supplemented with wheat germ. Int. J. Food Sci. Technol..

[139] Petrović J., Fišteš A., Rakić D., Pajin B., Lončarević I., Šubarić D. (2015). Effect of defatted wheat germ content and its particle size on the rheological and textural properties of the cookie dough. J. Texture Stud..

[140] Youssef H.M.K.E. (2015). Assessment of gross chemical composition, mineral composition, vitamin composition and amino acids composition of wheat biscuits and wheat germ fortified biscuits. Food Nutr. Sci..

[141] Călinoiu L.F., Vodnar D.C. (2019). Thermal processing for the release of phenolic compounds from wheat and oat bran. Biomolecules.

[142] Jackowski M., Niedźwiecki Ł., Jagiełło K., Uchańska O., Trusek A. (2020). Brewer’s Spent Grains—Valuable Beer Industry By-Product. Biomolecules.

[143] Görgüç A., Özer P., Yılmaz F.M. (2020). Microwave-assisted enzymatic extraction of plant protein with antioxidant compounds from the food waste sesame bran: Comparative optimization study and identification of metabolomics using LC/Q-TOF/MS. J. Food Process. Preserv..

[144] Bacha E.G. (2022). Response Surface Methodology Modeling, Experimental Validation, and Optimization of Acid Hydrolysis Process Parameters for Nanocellulose Extraction. S Afr. J. Chem. Eng..

[145] Ideia P., Sousa-Ferreira I., Castilho P.C. (2020). A Novel and Simpler Alkaline Hydrolysis Methodology for Extraction of Ferulic Acid from Brewer’s Spent Grain and Its (Partial) Purification through Adsorption in a Synthetic Resin. Foods.

[146] Macias-Garbett R., Serna-Hernández S.O., Sosa-Hernández J.E., Parra-Saldívar R. (2021). Phenolic Compounds From Brewer’s Spent Grains: Toward Green Recovery Methods and Applications in the Cosmetic Industry. Front. Sustain. Food Syst..

[147] Guido L.F., Moreira M.M. (2017). Techniques for Extraction of Brewer’s Spent Grain Polyphenols: A Review. Food Bioprocess Technol..

[148] Rebolleda S., José M.L.G.S., Sanz M.T., Beltrán S., Solaesa Á.G. (2020). Bioactive Compounds of a Wheat Bran Oily Extract Obtained with Supercritical Carbon Dioxide. Foods.

[149] Zheng X., Zhang R., Liu C. (2015). Extraction and Antioxidant Activity of Phenolic Compounds from Wheat Bran Treated by Steam Explosion. Trop. J. Pharm. Res..

[150] Radenkovs V., Juhnevica-Radenkova K., Górnaś P., Seglina D. (2018). Non-Waste Technology through the Enzymatic Hydrolysis of Agro-Industrial by-Products. Trends Food Sci. Technol..

[151] Călinoiu L.F., Cătoi A.F., Vodnar D.C. (2019). Solid-State Yeast Fermented Wheat and Oat Bran as A Route for Delivery of Anti-oxidants. Antioxidants.

[152] Dynkowska W.M. (2019). Rye (*Secale Cereale* L.) Phenolic compounds as health-related factors. Plant Breed Seed Sci..

[153] Schmidt C.G., Cerqueira M.A., Vicente A.A., Teixeira J.A., Furlong E.B. (2015). Rice bran protein-based films enriched by phenolic extract of fermented rice bran and montmorillonite clay. CyTA J. Food.

[154] Wang N., Saleh A.S.M., Gao Y., Wang P., Duan Y., Xiao Z. (2019). Effect of protein aggregates on properties and structure of rice bran protein-based film at different pH. J. Food Sci. Technol..

[155] Fritsch C., Staebler A., Happel A., Márquez M.A.C., Aguiló-Aguayo I., Abadias M., Gallur M., Cigognini I.M., Montanari A., López M.J. (2017). Processing, valorization and application of bio-waste derived compounds from potato, tomato, olive and cereals: A review. Sustainability.

